# Same-Day Primary Care Referral Versus Usual Care for Patients With Elevated Blood Pressures Seen in a Preoperative Clinic

**DOI:** 10.7759/cureus.58401

**Published:** 2024-04-16

**Authors:** Shaunte Butler, Natalie Holt, Feng Dai, Catherine Quick, Jeffrey D Kravetz, Albert C Perrino, Robert B Schonberger

**Affiliations:** 1 Anesthesiology and Critical Care, Massachusetts General Hospital, Harvard Medical School, Boston, USA; 2 Anesthesiology, Indian Health Service, Aberdeen, USA; 3 Statistics, Yale School of Medicine, New Haven, USA; 4 Anesthesiology, Veterans Affairs Connecticut Healthcare, West Haven, USA; 5 Primary Care, Yale School of Medicine, Veterans Affairs Connecticut Healthcare, West Haven, USA; 6 Anesthesiology, Yale School of Medicine, Veterans Affairs Connecticut Healthcare System, West Haven, USA; 7 Anesthesiology, Yale School of Medicine, New Haven, USA

**Keywords:** veterans health, academic anesthesiology, quality improvement, hypertension, preoperative clinic

## Abstract

Background

While several studies have suggested that anesthesia and surgical care episodes provide an opportunity to improve the diagnosis and treatment of hypertension, few studies have implemented and tested pragmatic care coordination efforts for this population. The present study aimed to examine the effects of same-day primary care referral vs. usual care on outpatient hypertension treatment among patients with elevated preoperative clinic blood pressure (BP).

Methodology

With institutional review board approval of the project as a quality improvement (QI) initiative not requiring consent, we conducted a prospective QI project comparing same-day preoperative primary care referral vs. usual care within comparable cohorts of US Veterans presenting to a preoperative evaluation clinic with elevated BP for whom treatment assignment was based on prior primary care clinic affiliation. Outpatient BP, antihypertensive medications, and antihypertensive dosages at the initial visit and for one year after the initial preoperative clinic visit were followed in the electronic health record.

Results

Between June 1, 2018, and June 1, 2019, one of the two on-site primary care groups (Firm A) at our facility agreed to accommodate same-day BP referrals. Patients in the second primary care group received standard preoperative care (Firm B). Charts for the pseudo-randomized cohort of Firm A and B patients were compared after 12 months to assess for changes in BP and hypertension treatment. Firm A and B patients were similar in demographics. Overall, 68 (91%) Firm A patients were correctly referred for primary care appointments. Moreover, 28 of 68 (41.2%) patients adhered to the same-day referral recommendation, with the remainder declining to attend the primary care visit. BPs were similar between Firm A and Firm B groups at 3, 6, 9, and 12 months post-intervention. Firm A adherent patients (i.e., those attending the referral) received hypertension treatment intensification sooner than Firm A non-adherent and Firm B patients (median (interquartile range) days to intensification = 21 (0.5-103.5) vs. 154 (45.5-239) and 170 (48-220), respectively; p = 0.038 and p = 0.048, respectively).

Conclusions

Our protocol achieved a high degree of same-day primary care referral (91%) in hypertensive patients presenting at the preoperative clinic. Although this limited study did not demonstrate improved BP control in patients who received same-day primary care, this group did show increased rates of rapid treatment intensification which may infer improved long-term health outcomes. Further work examining logistical barriers to patients attending same-day referrals is warranted.

## Introduction

Hypertension remains the largest modifiable risk factor for global disease morbidity and mortality [1]. Previous studies have shown that a decrease in as little as 2 mmHg systolic blood pressure (SBP) correlates to a 25% decrease in stroke and a 10% decrease in major cardiovascular events, while a decrease in as little as 2 mmHg diastolic blood pressure (DBP) correlates to a 12% reduction in the risk of major cardiovascular events [2,3]. Despite the importance of hypertension control for longitudinal cardiovascular health, in 2014, approximately 12.8% of adults in the United States had uncontrolled hypertension, and among those with hypertension, 15.9% remained unaware of their condition [4,5].

Several investigations have suggested that the perioperative care episode is an opportunity to identify poorly controlled hypertension in adults [6-9], and a recent study showed that elevated BP of >140/90, >150/95, and >160/100 mmHg, as measured at a preoperative clinic, yielded positive predictive values for truly elevated home BP of 84.1% (95% confidence interval (CI) = 0.78-0.89), 87.5% (95% CI = 0.81-0.92), and 94.6% (95% CI = 0.87-0.99), respectively [10]. However, it is not yet known what preoperative clinic interventions may result in improved hypertension outcomes.

In this context, we designed and herein report the results of a quality improvement (QI) project at a local Veterans Affairs preoperative clinic that provided same-day primary care referrals for patients found to have elevated BP during their preoperative clinic visits. A rigorous, pseudo-randomized approach to assessing this QI intervention vs. usual care was made possible by the design of our local West Haven Veterans Affairs Medical Center’s (WHVA) primary care clinics. WHVA has two panels of primary care patients (Firm A and Firm B), and Veterans are assigned to one or the other panel based on an arbitrary ordering of their enrollment along with the available clinic census. The arbitrary assignment of patients to Firm A vs. Firm B intersected with the willingness of Firm A clinicians to accommodate the logistical challenge of same-day, elective hypertension referral following preoperative evaluations. Qualifying patients from Firm A were thus referred for same-day primary care BP management, while a highly similar cohort of patients from Firm B was handled in accordance with the standard of care, including counseling about their elevated BP and a recommendation to follow up with their primary care physician. The resulting unbiased, pseudo-randomized BP referral program based on Firm A vs. Firm B primary care assignment was presented to the relevant institutional review board (IRB) and was approved as a QI project without the requirement of informed consent. The primary outcomes of the QI intervention included (a) changes in clinic-measured BP and (b) time until BP treatment intensification as identified in the electronic health record (EHR) in the year following preoperative evaluation.

## Materials and methods

The protocol for the project was presented to the WHVA Institutional Review Board where it was approved as a QI project without the requirement of informed consent. This manuscript adheres to the applicable SQUIRE guidelines [11].

Inclusion and exclusion criteria

Patients who presented for anesthesia preoperative evaluation at the WHVA between June 1, 2018, and June 1, 2019, and who were receiving primary care at the WHVA were eligible for this QI program. As part of the usual anesthesia preoperative evaluation, patients checking into the preoperative clinic have their BP measured while in a seated position using an automated oscillometric sphygmomanometer. Patients qualified for the present QI initiative if they had at least two BP readings in the preoperative clinic that showed an SBP ≥140 mmHg or a DBP ≥90 mm Hg, corresponding to the American Heart Association guidelines as stage 2 hypertension [12]. Within this qualifying group, all patients receiving primary care at the WHVA were eligible without further exclusions.

Intervention

As described above, patients at the WHVA are assigned in an arbitrary, pseudo-random fashion to one of two primary care panels, termed Firm A or Firm B. As the result of a cooperative arrangement with Firm A clinicians, hypertensive preoperative clinic patients from Firm A with elevated BP were referred for a same-day visit for BP evaluation and management, while patients in Firm B received the usual standard of care, including counseling regarding elevated BP and recommendations for such patients that they make a follow-up appointment with their primary care provider. Due to the arbitrary enrollment of patients into Firm A vs. Firm B, the resulting population of referred and unreferred patients would be expected to provide relatively unbiased, pseudo-randomized populations to compare the two care pathways.

Data collection

Hypertensive patients were followed via the EHR for 12 months after their preoperative appointment to collect information regarding their BP, hypertension treatment, and clinic visits.

Specific data collected for the QI initiative included age at the time of the initial preoperative visit, sex, race (as identified by the patient), Hispanic ethnicity (as identified by the patient), body mass index (calculated as the weight of the patient in kilograms divided by the square of the height in meters), history of smoking, previous history of and treatment for hypertension, patient’s assigned primary care clinic, the subspecialty of the surgeon performing the planned procedure that led to the preoperative visit, observed preoperative clinic BP, Charlson Comorbidity Index at the time of the preoperative visit, baseline outpatient SBP and DBP before the preoperative visit, and whether patients were referred to and attended a same-day primary care appointment for BP consultation.

Outcome assessment

For outcome ascertainment, data included the following: whether patients presented for a same-day clinic referral after the preoperative visit, outpatient mean SBP and DBP at 3-6, 6-9, and 9-12-month time-periods after the initial preoperative visit, number of days from the preoperative visit to the next primary care visit, and number of days until antihypertensive treatment intensification, defined as any increase in the dose or number of prescribed BP medications.

Statistical analyses

For descriptive variables, mean and standard deviation (SD) or median and interquartile range (IQR) are reported for continuous variables as appropriate, and frequency (percentage) is presented for categorical variables.

For changes in BP, we planned a difference-in-difference analysis of mean SBP and DBP, at 3-6, 6-9, and 9-12 months after the initial preoperative visit based on intention-to-treat. Differences from baseline at the three time points between the two groups were analyzed within a mixed-effects model with repeated measures analysis, in which an unstructured covariance matrix was specified to account for the within-subject correlations. Group, time (3-6, 6-9, and 9-12 months), group and time interaction, and baseline value of SBP (or DBP) were included as fixed effects in the model. The least squares mean of SBP (DBP) changes and 95% CI were estimated at each time point for both groups, and the between-group differences and 95% CI were calculated.

Further exploratory analyses

On preliminary review of the data, it was clear that only a minority of Firm A patients were adherent to the QI intervention (i.e., they accepted and presented for the same-day primary care referral). Thus, further post hoc exploratory analyses were conducted on subgroups to compare Firm A adherent patients (i.e., those who attended the referral appointment) with Firm A non-adherent patients and Firm B patients. For analyses of these three subgroups, median (IQR) days to the next primary care visit and days to antihypertensive treatment intensification are reported, and these were assessed for difference using pairwise Wilcoxon rank-sum tests.

Statistical software SAS version 9.4 (Cary, NC) was used for all analyses. All the tests were two-sided, and a p-value of less than 0.05 was considered to suggest statistical significance. As this project was a QI endeavor without a primary inferential research aim, no limit was placed on the number of included participants, and an a priori power analysis was not performed.

## Results

A total of 1,272 patients were evaluated at the WHVA preoperative clinic between June 1, 2018, and June 1, 2019. Of this total, 306 (24.1%) belonged to Firm A, 230 (18.1%) belonged to Firm B, and 736 (57.8%) belonged to primary care clinics outside of the WHVA.

**Figure 1:**
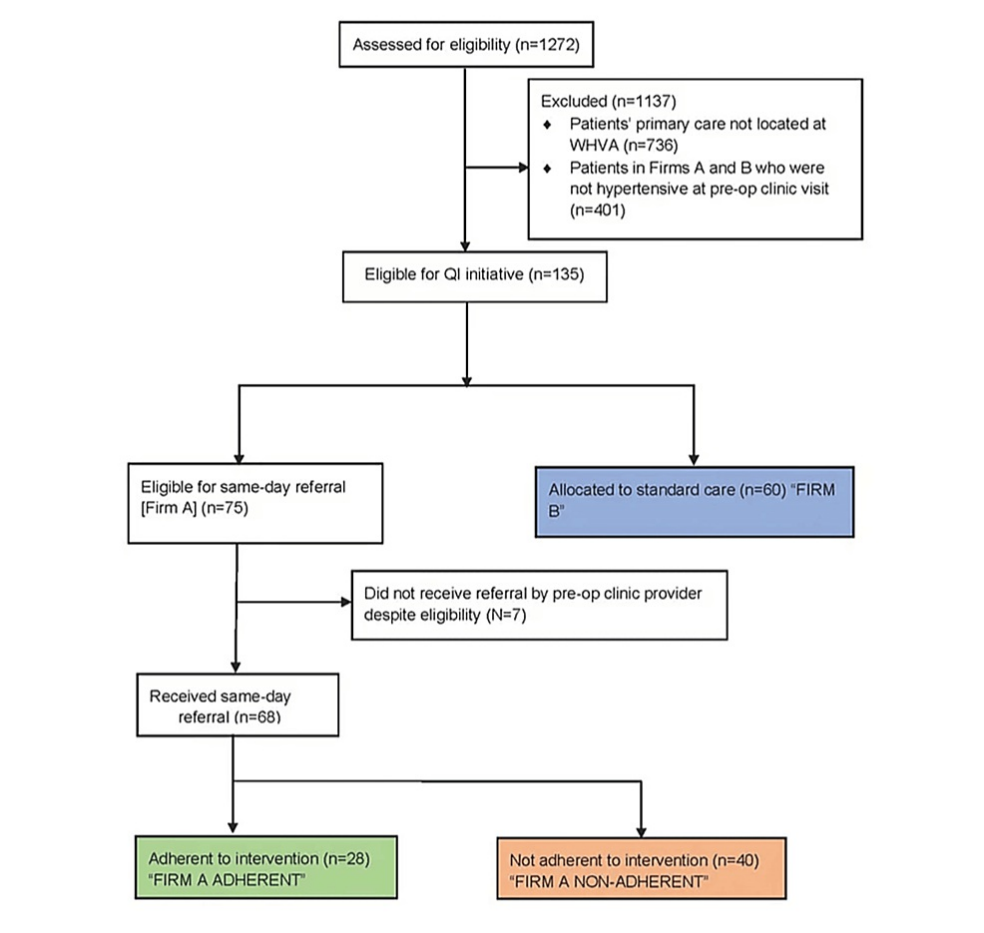
CONSORT diagram illustrating the formation of the analytic dataset.

Of the 536 patients receiving primary care locally at the WHVA, 75 patients in Firm A and 60 patients in Firm B demonstrated clinic BP ≥140/90mmHg. Of the patients eligible for the QI intervention, 68 (91%) of eligible Firm A patients and 0 (0%) Firm B patients were referred for same-day primary care visits. Of the 68 referred patients from Firm A, 28 (41%) adhered to the same-day referral intervention. See Figure 1 (CONSORT diagram) for a description of the included cohort.

Between Firm A and Firm B patients, there were no significant differences in age, sex, race, Hispanic ethnicity, BMI, Charlson Comorbidity Index, history of smoking, history of hypertension, percent with hypertension at the preoperative clinic, number of antihypertensive medications at the time of the preoperative visit, first and second SBP at the preoperative clinic, first and second DBP readings at the preoperative clinic, or SBP average before the initial preoperative visit (see Table 1). Hypertensive patients in Firm A had a mean prior DBP of 76.3 compared to hypertensive patients in Firm B who had a mean prior DBP of 72.7.

**Table 1:** Patient demographics and baseline characteristics. Data are presented as mean (standard deviation), median (interquartile range: p25-p75), or n (%) as relevant. HTN = hypertension; BMI = body mass index; SBP = systolic blood pressure; DBP = diastolic blood pressure; BP = blood pressure

Characteristics	Firm A (N = 306)	Firm B (N = 230)	Total (N = 536)	P-value
Age (in years)	66.75 (11.23)	68.10 (11.17)	67.33 (11.21)	0.17
Male sex	300 (98%)	230 (100%)	530 (99%)	0.033
Race				0.17
White	230 (75.2%)	192 (83.5%)	422 (78.7%)	
Black	65 (21.2%)	31 (13.5%)	96 (17.9%)	
Native American	1 (0.3%)	1 (0.4%)	2 (0.4%)	
Asian	1 (0.3%)	0 (0%)	1 (0.2%)	
Other	9 (3%)	6 (2.6%)	15 (2.8%)	
Hispanic ethnicity	14 (5%)	14 (6%)	28 (5%)	0.44
BMI (kg/m^2^)	30.12 (5.74)	30.67 (6.04)	30.36 (5.87)	0.28
Charlson Comorbidity Index	4.72 (2.40)	4.78 (2.40)	4.75 (2.40)	0.77
Previous history of HTN	242 (79%)	168 (73%)	410 (76%)	0.1
History of smoking				0.97
Current	59 (19%)	46 (20%)	105 (20%)	
Past	180 (59%)	135 (59%)	315 (59%)	
Never	67 (22%)	49 (21%)	116 (22%)	
SBP average before preoperative	134.97 (14.12)	132.08 (14.79)	133.73 (14.46)	0.022
DBP average before preoperative	73.40 (9.12)	70.47 (9.38)	72.14 (9.34)	<0.001
BP medications at visit	1.0 (0.0–2.0)	1.0 (0.0–2.0)	1.0 (0.0–2.0)	0.94
SBP reading #1 at preoperative	133.38 (19.10)	133.87 (17.75)	133.59 (18.52)	0.76
SBP reading #2 at preoperative	130.19 (17.38)	131.58 (16.72)	130.79 (17.10)	0.35
DBP reading #1 at preoperative	71.19 (10.39)	71.73 (10.53)	71.42 (10.45)	0.55
DBP reading #2 at preoperative	70.27 (10.23)	71.08 (10.06)	70.62 (10.16)	0.36
Hypertensive at preoperative	74 (24%)	61 (27%)	135 (25%)	0.54
Same-day primary care referral	68 (22%)	0 (0%)	67 (13%)	<0.001
Type of surgery				0.93
Cardiac, thoracic, and vascular	30 (10%)	22 (10%)	52 (10%)	
Other	276 (90%)	208 (90%)	484 (90%)	

Difference-in-difference analysis

There were no significant differences in changes in SBP or DBP from baseline between Firm A and Firm B patients within the intention-to-treat analysis. Firm A vs. Firm B change in SBP at 3-6 months was -2.50 vs. -1.69 mmHg (p = 0.78), at 6-9 months was -4.68 vs. -3.92 mmHg (p = 0.80), and at 9-12 months was -2.31 vs. -4.27 mmHg (p = 0.51). Firm A vs. Firm B change in DBP at 3-6 months was -1.34 vs. -0.30 mmHg (p = 0.50), at 6-9 months was -1.55 vs. -1.34 mmHg (p = 0.90), and at 9-12 months was -1.75 vs. -1.72 mmHg (p = 0.98) (see Table 2). In a post hoc per-protocol analysis comparing Firm A patients who adhered to the recommendation with Firm A non-adherent and Firm B patients, there were no significant differences in changes in SBP or DBP from baseline at any of the time intervals.

**Table 2:** Comparison of changes in SBP and DBP from baseline among patients presenting to the preoperative clinic with hypertension. Data are presented as mean (SD). SBP = systolic blood pressure; DBP = diastolic blood pressure; mmHg = millimeters of mercury

Time	Firm A (N = 75)	Firm B (N = 60)	Difference (95% CI)	P-value
SBP changes (mmHg)				
3–6 months	-2.50 (-6.25 to 1.44)	-1.69 (-5.91 to 2.54)	-0.82 (-6.47 to 4.84)	0.78
6–9 months	-4.68 (-8.37 to 0.99)	-3.02 (-8.40 to 0.55)	-0.75 (-6.56 to 5.05)	0.80
9–12 months	-2.31 (-6.18 to 1.57)	-4.27 (-8.66 to 0.11)	1.97 (-3.89 to 7.82)	0.51
DBP changes (mmHg)				
3–6 months	-1.34 (-3.33 to 0.65)	-0.30 (-2.55 to 1.96)	-1.-4 (-4.06 to 1.97)	0.50
6–9 months	-1.55 (-3.52 to 0.42)	-1.34 (-3.72 to 1.03)	-0.32 (-3.30 to 2.89)	0.90
9–12 months	-1.75 (-3.80 to 0.29)	-1.72 (-4.05 to 0.62)	-0.03 (-3.14 to 3.08)	0.98

Primary care follow-up and medication intensification analysis

Patients in Firm A had a median of seven days to their next primary care visit compared to Firm B patients who had a median of 39.5 days to their next primary care visit, a difference that demonstrated statistical significance (p = 0.007) (Table 1). On survival analysis using a univariate Cox regression model, Firm A patients were 61% more likely to have a primary care visit, within the 12-month follow-up period, than Firm B patients (hazard ratio (HR) = 1.61 95% CI = 1.13-2.30; p = 0.008).

Post hoc analysis of adherent vs. non-adherent patients

**Figure 2:**
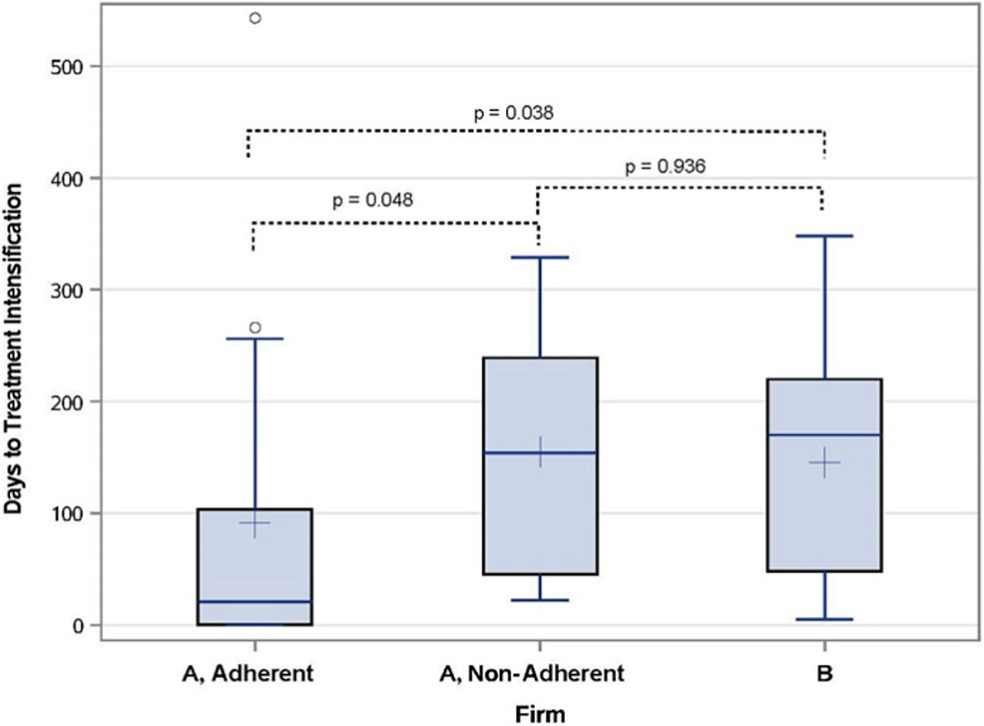
Days to antihypertensive treatment intensification. Boxplots comparing days to treatment intensification between Firm A adherent, Firm A non-adherent, and Firm B patients. Boxes represent the 25th-75th percentile with whiskers to 1.5 times the interquartile range and outliers marked as hollow circles. Lines inside boxes represent the median, and plus signs inside denote the mean.

The median time to antihypertensive medication intensification in Firm A adherent patients was 21 days compared to a median of 154 days among Firm A non-adherent patients (p = 0.048) and 170 days among Firm B patients (p = 0.038) (Figure 2). The post hoc analysis further demonstrated that patients who adhered to the same-day referral intervention were more likely to receive BP medication intensification at 3, 7, and 10 months post-evaluation than either Firm A non-adherent or Firm B patients (36% vs. 13% vs. 10% at 3 months, p = 0.009; 50% vs. 15% vs. 18% at 6 months, p = 0.001; and 50% vs. 23% vs. 27% at 9 months, p = 0.04).

## Discussion

In the present analysis, we describe the effects of an anesthesiologist-initiated QI program designed to provide same-day referral to primary care providers for a sample of preoperative clinic patients with elevated BP. The project demonstrated a high degree of willingness by preoperative clinic clinicians to promote the intervention, with 91% (n = 68) of eligible hypertensive patients being correctly referred for same-day primary care visits. This finding compares favorably with the findings of a recent study from South Africa where the adherence rate for anesthesiologists to diagnose and manage uncontrolled hypertension in their preoperative clinic was 84% [12]. In contrast with provider willingness, our QI project demonstrated only modest patient willingness to accept same-day primary care referral, with only 41% of referred patients complying with the intervention.

Although clinicians did not systematically collect data to understand patients’ hesitancy to attend the same-day referral intervention, several clinicians recalled that patients frequently reported logistical reasons that prevented their attendance at the BP clinic. Even though the primary care clinic was in the same building as the preoperative clinic, an additional unplanned medical visit frequently conflicted with patients’ schedules, and it is possible that on-site primary care within the preoperative clinic may result in better adherence during future QI interventions. Additional dedicated studies are needed to better understand the reasons for patients’ low adherence to same-day primary care visits.

Regarding the potentially positive effects of the QI program, while we did not detect significant improvements in BP among patients who complied with the intervention, we did find significantly increased rates of BP treatment intensification among adherent Firm A patients compared to the other groups within three, six, and nine months. This improvement in the timing of treatment intensification may be important for the long-term health outcomes of this population, as previous investigations have implicated treatment inertia as an important and modifiable cause of poorly controlled BP [13-15]. Treatment inertia is a well-known problem in successfully managing hypertension, and in a study by Oliveria et al., antihypertensive treatment was initiated or intensified in only 38% of primary care visits of poorly controlled hypertensive patients, despite these patients having a documented elevated BP for at least six months before the visit [16-19]. Of note, a limitation of our analysis is that we considered any new treatment as treatment intensification without differentiating between zero treatment to some treatment vs. some treatment to more treatment. It has been suggested that going from no treatment to some treatment may be more impactful for long-term health, and it is possible that the QI intervention was particularly beneficial to this subgroup who were treatment-naïve before our intervention.

Despite improvements regarding treatment inertia, the finding that BP did not improve in our QI cohort may reflect the limited power of our sample size, as well as the well-known difficulty in achieving long-term BP control among patients with consistently elevated BP. Hypertension is much easier to diagnose than it is to control, and it would not be surprising to find that a single additional primary care appointment may not be sufficient to strongly alter BP trajectories within a relatively small sample of patients in the course of a single year. Consistent with this hypothesis, the American College of Cardiology and American Heart Association recommend that patients with stage 2 hypertension have their BP reassessed monthly when BP is persistently not at goal [20]. Further studies are needed to assess if same-day referral with more frequent monitoring of poorly controlled hypertensive patients in the preoperative clinic setting may lead to significant BP changes or improvements in hypertension-associated morbidity across longer time scales.

Among the notable strengths of the present report is that the structure of the QI protocol appears to have created highly comparable groups, as there were no significant differences observed between the referral group (Firm A) and the usual care group (Firm B) in age, sex, race, Hispanic ethnicity, BMI, Charlson Comorbidity Index, hypertension history, smoking status, and prior SBP average. While there was a difference in the mean DBP before preoperative visits between the two groups, our analysis sought to account for that by performing analyses on the difference-in-difference between the groups at all time points, rather than simply analyzing the differences in absolute BP between the groups.

Additional limitations of our QI project should be noted. One limitation of our analysis is that it was likely underpowered to detect clinically significant changes in BP. While we intended to include all patients who arrived at the preoperative clinic throughout a one-year period, once patients who did not have qualifying hypertension measurements on preoperative visits and patients whose primary care clinic was not at the WHVA were excluded, we were only left with 75 patients in the intervention group and 60 patients in the usual care group. As noted earlier, a change in as little as 2 mmHg SBP and 1 mmHg DBP can be enough to significantly decrease the risk of major cardiovascular events, effect sizes that were clearly too small to have been seen within our small cohort [2,3]. Another limitation of our QI project concerns the issue of generalizability, as the patient population we studied was not reflective of national demographics regarding important characteristics, including age, gender, race, and ethnicity [21]. Nevertheless, although our study was limited to US Veterans, the VA medical system constitutes the largest unified healthcare system in the US and no doubt contains ample opportunities to improve public health by enhancing the care of hypertensive patients who present for surgery within the VA system [22].

## Conclusions

Within a QI program that initiated same-day primary care referral of hypertensive veterans presenting for presurgical evaluations, our protocol achieved a high degree of same-day primary care referral (91%) in hypertensive patients presenting at the preoperative clinic. Although this limited study did not demonstrate improved BP control in patients who received same-day primary care, this group did show increased rates of rapid treatment intensification which may infer improved long-term health outcomes. Further work examining logistical barriers to patients attending same-day referrals is warranted.

Additional efforts to define optimal ways of managing longitudinal care of hypertensive surgical patients in the context of the preoperative clinic may lead to significant improvements in morbidity despite the challenging and episodic nature of the preoperative care environment.

## References

[1] Lim SS, Vos T, Flaxman AD (2012). A comparative risk assessment of burden of disease and injury attributable to 67 risk factors and risk factor clusters in 21 regions, 1990-2010: a systematic analysis for the Global Burden of Disease Study 2010. Lancet.

[2] Turnbull F (2003). Effects of different blood-pressure-lowering regimens on major cardiovascular events: results of prospectively-designed overviews of randomised trials. Lancet.

[3] Verdecchia P, Gentile G, Angeli F, Mazzotta G, Mancia G, Reboldi G (2010). Influence of blood pressure reduction on composite cardiovascular endpoints in clinical trials. J Hypertens.

[4] Sakhuja A, Textor SC, Taler SJ (2015). Uncontrolled hypertension by the 2014 evidence-based guideline: results from NHANES 2011-2012. J Hypertens.

[5] Paulose-Ram R, Gu Q, Kit B (2017). Characteristics of U.S. adults with hypertension who are unaware of their hypertension, 2011-2014. NCHS Data Brief.

[6] Drummond JC, Blake JL, Patel PM, Clopton P, Schulteis G (2013). An observational study of the influence of "white-coat hypertension" on day-of-surgery blood pressure determinations. J Neurosurg Anesthesiol.

[7] Schonberger RB (2014). Ideal blood pressure management and our specialty. J Neurosurg Anesthesiol.

[8] Schonberger RB, Burg MM, Holt N, Lukens CL, Dai F, Brandt C (2012). The relationship between preoperative and primary care blood pressure among veterans presenting from home for surgery: is there evidence for anesthesiologist-initiated blood pressure referral?. Anesth Analg.

[9] Schonberger RB, Dai F, Brandt CA, Burg MM (2015). Balancing model performance and simplicity to predict postoperative primary care blood pressure elevation. Anesth Analg.

[10] Schonberger RB, Nwozuzu A, Zafar J (2018). Elevated preoperative blood pressures in adult surgical patients are highly predictive of elevated home blood pressures. J Am Soc Hypertens.

[11] Ogrinc G, Davies L, Goodman D, Batalden P, Davidoff F, Stevens D (2016). SQUIRE 2.0 (Standards for QUality Improvement Reporting Excellence): revised publication guidelines from a detailed consensus process. BMJ Qual Saf.

[12] Pfister CL, Govender S, Dyer RA (2020). A multicenter, cross-sectional quality improvement project: the perioperative implementation of a hypertension protocol by anesthesiologists. Anesth Analg.

[13] Ferrari P (2009). Reasons for therapeutic inertia when managing hypertension in clinical practice in non-Western countries. J Hum Hypertens.

[14] Harle CA, Harman JS, Yang S (2013). Physician and patient characteristics associated with clinical inertia in blood pressure control. J Clin Hypertens (Greenwich).

[15] Kjeldsen SE, Julius S, Dahlöf B, Weber MA (2015). Physician (investigator) inertia in apparent treatment-resistant hypertension - insights from large randomized clinical trials. Lennart Hansson Memorial Lecture. Blood Press.

[16] Hicks LS, Fairchild DG, Horng MS, Orav EJ, Bates DW, Ayanian JZ (2004). Determinants of JNC VI guideline adherence, intensity of drug therapy, and blood pressure control by race and ethnicity. Hypertension.

[17] Berlowitz DR, Ash AS, Hickey EC, Friedman RH, Glickman M, Kader B, Moskowitz MA (1998). Inadequate management of blood pressure in a hypertensive population. N Engl J Med.

[18] Asai Y, Heller R, Kajii E (2002). Hypertension control and medication increase in primary care. J Hum Hypertens.

[19] Oliveria SA, Lapuerta P, McCarthy BD, L'Italien GJ, Berlowitz DR, Asch SM (2002). Physician-related barriers to the effective management of uncontrolled hypertension. Arch Intern Med.

[20] Whelton PK, Carey RM, Aronow WS (2018). 2017 ACC/AHA/AAPA/ABC/ACPM/AGS/APhA/ASH/ASPC/NMA/PCNA guideline for the prevention, detection, evaluation, and management of high blood pressure in adults: a report of the American College of Cardiology/American Heart Association Task Force on Clinical Practice Guidelines. Hypertension.

[21] Fryar CD, Ostchega Y, Hales CM, Zhang G, Kruszon-Moran D (2017). Hypertension prevalence and control among adults: United States, 2015-2016. NCHS Data Brief.

[22] Morgan RO, Teal CR, Reddy SG, Ford ME, Ashton CM (2005). Measurement in Veterans Affairs Health Services Research: veterans as a special population. Health Serv Res.

